# A ^18^F-FDG PET/CT-based deep learning-radiomics-clinical model for prediction of cervical lymph node metastasis in esophageal squamous cell carcinoma

**DOI:** 10.1186/s40644-024-00799-0

**Published:** 2024-11-12

**Authors:** Ping Yuan, Zhen-Hao Huang, Yun-Hai Yang, Fei-Chao Bao, Ke Sun, Fang-Fang Chao, Ting-Ting Liu, Jing-Jing Zhang, Jin-Ming Xu, Xiang-Nan Li, Feng Li, Tao Ma, Hao Li, Zi-Hao Li, Shan-Feng Zhang, Jian Hu, Yu Qi

**Affiliations:** 1 Thoracic Surgery, The First Affiliated Hospital of Zhengzhou University, Zhengzhou, Henan province China; 2 Surgical Oncology, Shanghai Chest Hospital, Shanghai Jiao Tong University, Shanghai, China; 3 Thoracic Surgery, Shanghai Chest Hospital, Shanghai Jiao Tong University, Shanghai, China; 4 Department of nuclear medicine and radiology, The First Affiliated Hospital of Zhengzhou University, Zhengzhou, Henan province China; 5 School of Basic Medical Science, Zhengzhou University, Zhengzhou, Henan province China; 6 Thoracic Surgery, The First Affiliated Hospital of Zhejiang University, Hangzhou, Zhejiang province China; 7 School of Artiffcial Intelligence, Sun Yat-sen University, Zhuhai, Guangdong province China

**Keywords:** Radiomics, Deep learning, Medical images analysis, PET/CT, Esophageal squamous cell carcinoma, Cervical lymph node metastasis, Prediction model

## Abstract

**Background:**

To develop an artificial intelligence (AI)-based model using Radiomics, deep learning (DL) features extracted from ^18^F-fluorodeoxyglucose (^18^F-FDG) Positron emission tomography/Computed Tomography (PET/CT) images of tumor and cervical lymph node with clinical feature for predicting cervical lymph node metastasis (CLNM) in patients with esophageal squamous cell carcinoma (ESCC).

**Methods:**

The study included 300 ESCC patients from the First Affiliated Hospital of Zhengzhou University who were divided into a training cohort and an internal testing cohort with an 8:2 ratio. Another 111 patients from Shanghai Chest Hospital were included as the external cohort. For each sample, we extracted 428 PET/CT-based Radiomics features from the gross tumor volume (GTV) and cervical lymph node (CLN) delineated layer by layer and 256 PET/CT-based DL features from the maximum cross-section of GTV and CLN images We input these features into seven different machine learning algorithms and ultimately selected logistic regression (LR) as the model classifier. Subsequently, we evaluated seven models (Clinical, Radiomics, Radiomics-Clinical, DL-Clinical, DL-Radiomics, DL-Radiomics-Clinical) using Radiomics features, DL features and clinical feature.

**Results:**

The DL-Radiomics-Clinical (DRC) model demonstrated higher AUC of 0.955 and 0.916 compared to the other six models in both internal and external testing cohorts respectively. The DRC model achieved the highest accuracy among the seven models in both the internal and external test sets, with scores of 0.951 and 0.892, respectively.

**Conclusions:**

Through the combination of Radiomics features and DL features from PET/CT imaging and clinical feature, we developed a predictive model exhibiting exceptional classification capabilities. This model can be considered as a non-invasive method for predication of CLNM in patients with ESCC. It might facilitate decision-making regarding to the extend of lymph node dissection, and to select candidates for postoperative adjuvant therapy.

**Supplementary Information:**

The online version contains supplementary material available at 10.1186/s40644-024-00799-0.

## Background

Esophageal carcinoma (EC) has become the sixth leading cause of cancer-related deaths globally [1]. Esophageal squamous cell carcinoma (ESCC) is a major histologic subtype that is most prevalent in East Asian and Middle Eastern regions [2, 3]. The cancer-related mortality of ESCC is mainly attributed to high risk of lymphatic spread longitudinally along the lymphatic and nerve plexus [4], with reported incidence ranging from 20 to 40% depending on the location and tumor stage [5]. Cervical lymph node metastasis (CLNM) is defined as No. 100 (Superficial lymph nodes of the neck), No. 101 (Cervical paraesophageal lymph nodes), No. 102 (Deep cervical lymph nodes), No. 103 (Peripharyngeal lymph nodes), and No. 104 (Supraclavicular lymph nodes) lymph node metastasis according to the 11th edition of the Japanese Classification of Esophageal Cancer. It is common in ESCC patients with a prevalence of at least 20% [6, 7]. Even in superficial ESCC with malignant invasion limited to submucosa [8], the prevalence of occult CLNM still ranges from 1.3 to 13% [9–12].

CLNM not only affects the prognosis of ESCC patients but influences the choice of operative treatment, such as the selection of surgical procedure or lymph node dissection methods. Accurate assessment of CLNM in ESCC is of great significance governing procedure selection for early stage ESCC (i.e., endoscopic resection vs. radical esophagectomy), because candidates for endoscopic resection need cautious selection to avoid occult CLNM who ought to be referred to esophagectomy with lymphadenectomy, and determining the extend of lymphdenectomy for locally advanced ESCC (i.e., 2-field [thoracic-abdominal] versus 3-field [cervical-thoracic-abdominal] lymphdenectomy), which is under ongoing debate on balancing up-front 90-day mortality caused by additional surgical trauma with more-accurate staging and unproven long-term survival [13, 14]. Unfortunately, the first-line, non-invasive used methods for diagnosis of LNM in patients with EC are of limited capability and lead to inaccurate staging [15, 16], such as endoscopic ultrasound and contrast-enhanced computed tomography, both of which relies mainly on elementary morphology that is insufficient to distinguish metastasis from reactive hyperplasia or inflammation [17]. Therefore, a reliable non-invasive method to detect CLNM would have important implications for clinical decision support.

PET/CT using ^18^F-FDG enhances staging accuracy by adding metabolic data to anatomical insights [8, 18, 19]. However, relying solely on conventional parameters from initial scans for EC staging offers unstable sensitivity and specificity [20, 21]. Radiomics provided novel information by extracting and analyzing high-throughput quantitative images features [22]. Current research on its clinical use in EC primarily targets assessing radiotherapy and chemotherapy effectiveness [23–28], prognosis prediction [29, 30] and systematic LNM forecasting [31–34]. Deep learning (DL) presents a promising method for extracting comprehensive image features, both locally and globally. Convolutional neural networks (CNNs) serve as a quintessential approach for extracting high-dimensional numerical data from images by learning pertinent features from signal intensities [35]. Numerous studies [36, 37] have highlighted its impressive success in using DL in ESCC. These DL attributes are harvested using an extensive array of image filters. DL filter parameters are optimized using training images for superior classification results. Although DL’s data-driven feature extraction is considered more effective than radiomics’ handcrafted features [38], it’s uncertain if they are redundant or complementary in predicting CLNM in ESCC. Previous studies have demonstrated that models combining Radiomics and DL have superior predictive performance for LNM [39, 40]. This suggests that DL and Radiomics may complement each other in predicting LNM. Therefore, in this study, we explored the feasibility of joint modeling using DL features, Radiomics features, and clinical features. A recent review emphasized the importance of specifying the parameters in each modeling step during model development, which has positive significance for other researchers to replicate the study [41]. In this study, we reported the parameters selected during DL analysis and Radiomics analysis, striving to reproduce the details of the modeling process.

To the best of our knowledge, no studies has evaluated a PET/CT-based DL-Radiomics model for CLNM prediction in ESCC. We hypothesize that Radiomics and DL features are of independent potential value in predicating CLNM, and that a combination of Radiomics, DL and clinical features can improve to distinguish ESCC patients with and without CLNM. Therefore, we attempted to input the filtered DL features, Radiomics features, and clinical features to machine learning algorithms to establish a DL-Radiomics-Clinical (DRC) model, exploring whether this model can effectively predict CLNM in ESCC patients.

## Methods

### Study population

**Fig. 1:**
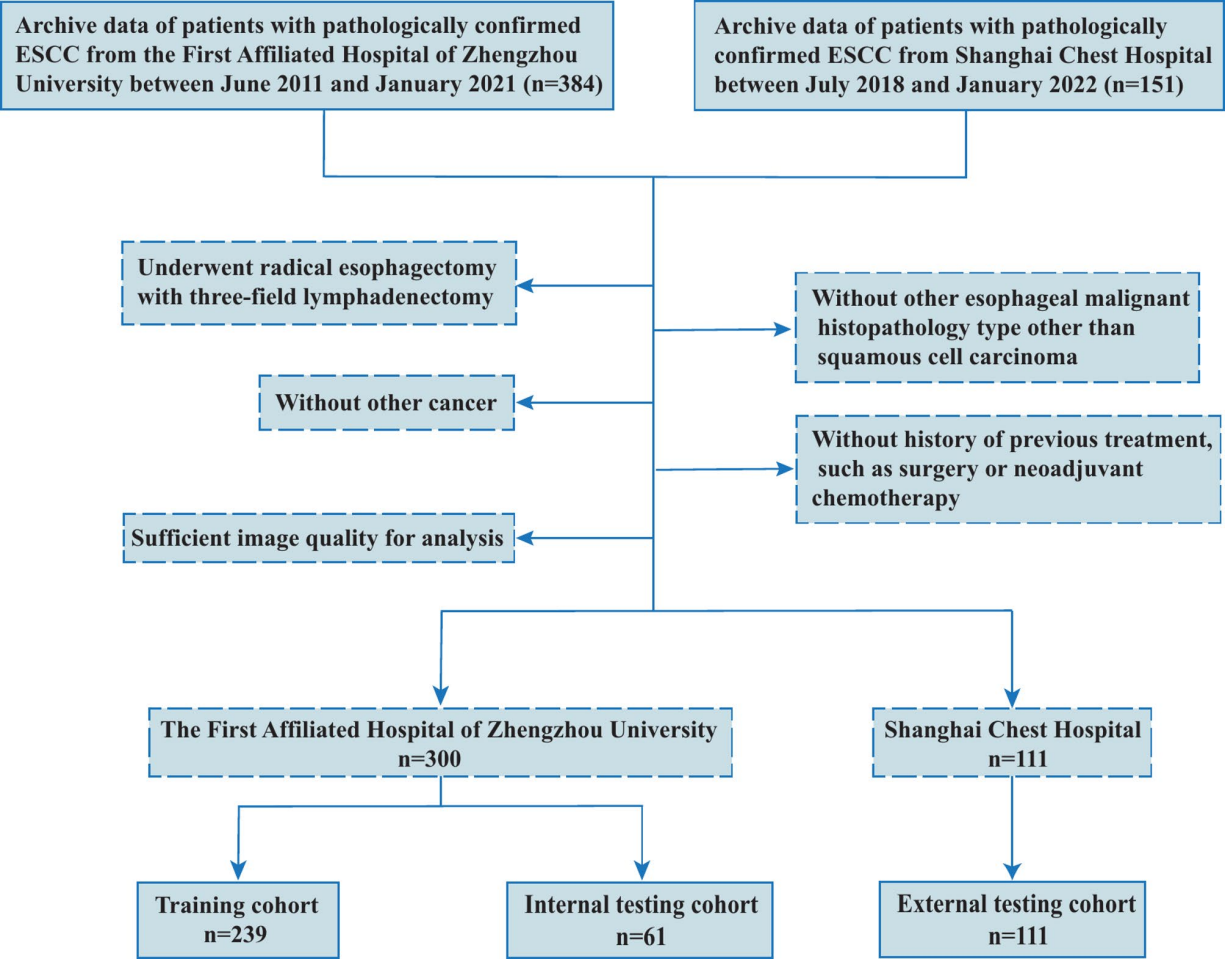
The flowchart of inclusion criteria of patients; ESCC, esophageal squamous cell carcinoma

Two ESCC patient cohorts were collected from the First Affiliated Hospital of Zhengzhou University (Center 1) and Shanghai Chest Hospital (Center 2). The inclusion criteria were follows: (1) underwent radical esophagectomy with 3-field lymphadenectomy; (2) without other histopathology type of EC; (3) without other primary cancer; (4) without history of neck surgery or neoadjuvant therapy; (5) have complete image data and adequate image quality (misalignment by respiratory motion were excluded) for analysis; Fig. 1 outlines patient selection, which leads to 411 participants. Among them, 300 patients from center 1 were divided into a training (*n* = 239) and internal testing cohort (*n* = 61) by an 8:2 ratio. The other 111 patients from center 2 were used to assessed model applicability. The study was approved by the Institutional Review Boards at the two centers adhering to ethical standards of the 1964 Helsinki Declaration and its later amendments.

Baseline clinicopathological data were obtained from hospital records, including age, sex, tumor length, tumor thickness, tumor differentiation, vascular infiltration, tumor location, T stage (the 8th edition of TNM classification), RLNM (recurrent laryngeal nerve lymph node metastasis), SLNM (supraclavicular lymph node metastasis), No.100 ~ No.103 LNM and thoracic or abdominal LNM. The explanation of tumor location is based on the lower margin of the azygos vein arch, which divides the lesion into the cervical/thoracic upper segment and the thoracic middle/lower segment (the 8th edition of TNM classification).

### Image preprocessing and tumor segmentation

**Fig. 2:**
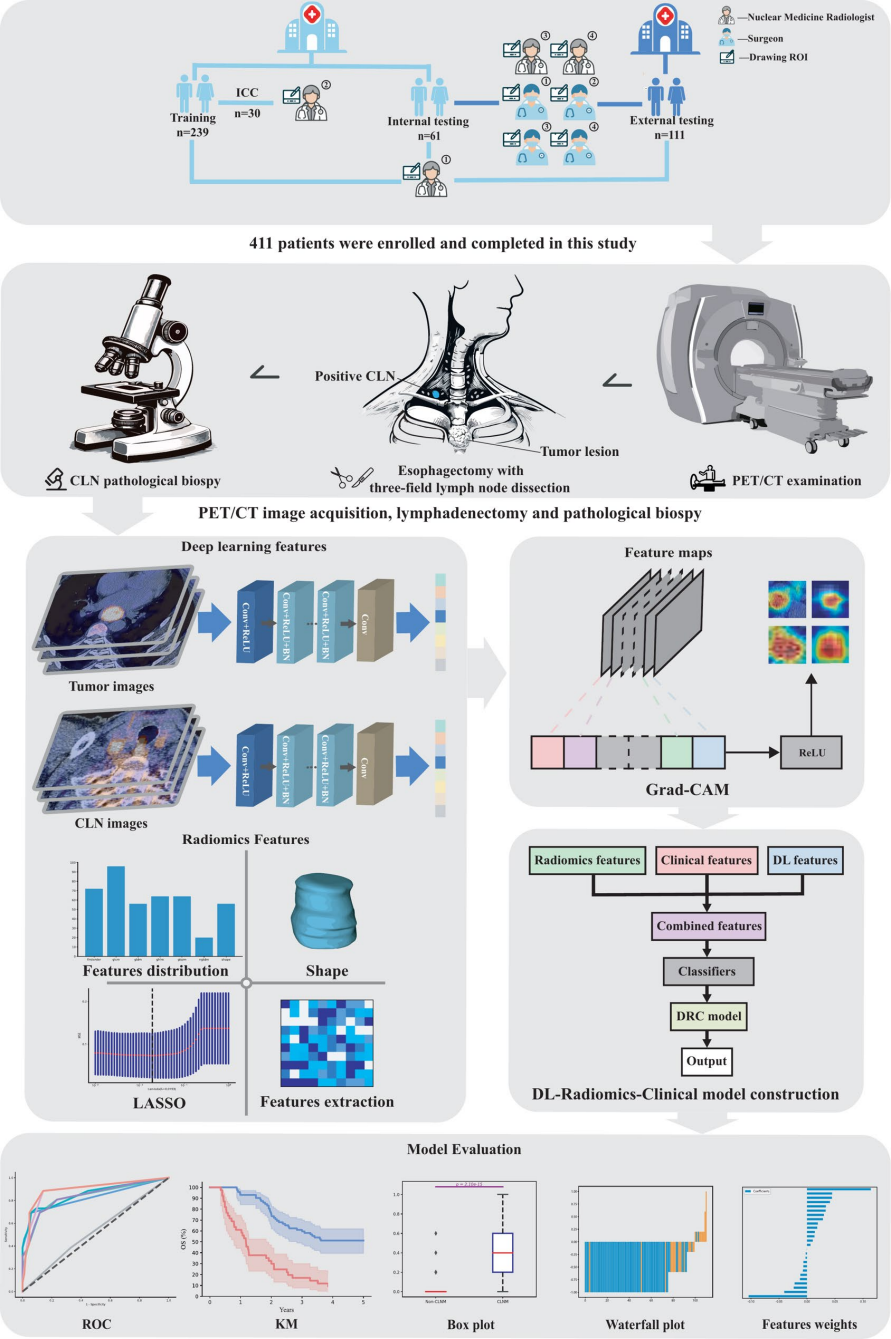
The workflow of this study. ROI, region of interest; DL, deep learning; CLN, cervival lymph node metastasis; LASSO, Least absolute shrinkage and selection operator; Grad-CAM, gradient-weighted class activation mapping; ROC, receiver operating characteristic; KM, kaplan-meier

Figure 2 shows the workflow of this study. Patients from both centers received ^18^F-FDG PET/CT scans using Siemens Biograph TruePoint 64-slice systems. ^18^F-FDG used in center 1 was synthesized via Sumitomo HM-20 cyclotron and CFL-100 module, achieving 98% radiochemical purity after quality control. ^18^F-FDG used in center 2 was produced and supplied by Shanghai Atom Kexin Pharmaceutical Co., Ltd. (Shanghai, China), with a pH value of ~ 7.0 and radiochemical purity of > 95%. All patients fasted for ≥ 6 h with blood glucose < 7.8 mmol/L pre-scan. Scanning commenced 1 h post ^18^F-FDG injection (0.10 ~ 0.15 mCi/kg based on patient weight). CT parameters were 120 kV and 40–120 mA, with a 0.8 s/rotation speed, followed by PET scans at 3 min/bed. The attenuation was corrected by CT and reconstructed by iterative method.

Before segmentation, a critical concern is that the original PET/CT images exhibit variations in voxel dimensions resulting from different CT equipment and setting. To resample the PET/CT images from both centers to a consistent resolution and size, SimpleITK package (version 2.0.2) was used to normalize the PET and CT images voxel size from 0.9765 × 0.9765 × 1.5 mm^3^ (Center 1) and 0.7246 × 0.7246 × 1 mm^3^ (Center 2) to 1 × 1 × 1 mm^3^. In addiation, the differences of CT image parameters were also minimized by standardizing CT image window width and window level to a same index. HUs were discretized to a bin width of 25 HU and the intensity of each image was truncated to a range of 0.5 to 99.5% points to minimize the side effects of voxel outliers.

Tumor segmentation was performed using 3D Slicer software (version 4.2.1). The normalized PET/CT images were inputted in DICOM format. A nuclear medicine radiologist (nuclear medicine radiologist 1, F.F.C) with over 5 years of experience manually delineated the regions of interest (ROIs) along the boundary of the tumor and CLN which is considered the most likely to be malignant based on its shape, size and SUV value via 3D Slicer. The ROI images contained the regions of whole tumor or cervical lymph node, which were manually cropped layer by layer from the raw PET/CT images. For DL, we extract the maximum cross-section of each ROI to serve as input for the convolutional neural network (CNN). During the process of segmentation, all delineations were reviewed by a senior nuclear medicine radiologist (K.S, nuclear medicine radiologist 2) particularly the uncertain ones.

### Radiomics features extraction

Radiomics features were extracted from the ROIs of tumor and CLN using Pyradiomics package (version 3.0.1). Supplementary Text 1 and 2 respectively showed the parameters used for Radiomics features extraction from CT images and PET images using Pyradiomics. Initially, we performed ROIs segmentation in a double-blind review process, conducted by nuclear medicine radiologist 1 who was informed about the diagnosis of ESCC, but was blinded to any clinical or pathological data. 30 random images were delineated (by K.S, nuclear medicine radiologist 2) and utilized to calculate intraclass correlation coefficients (ICCs) in order to ascertain the reliability and reproducibility of the Radiomics features. Radiomics characteristics having an ICC value of greater than 0.75 (indicating excellent consistency) were selected for feature extraction.

### DL features extraction

Resnet50, Alexnet, Googlenet, Mobilenet_V2, Mobilenet_V3, Shufflenet_V2 and Vgg16, pre-trained on the ImageNet datasets, were used for transfer learning. The PET/CT slice showing the maximum tumor or CLN ROI area was chosen as the original image and the resolution was normalized to 224 × 224 to adapt it to the network’s input size. DL features were extracted from each model’s penultimate layer for both internal and external cohorts. Feature dimensions were condensed using principal component analysis (PCA) to maintain the balance among features.

### Radiomics and DL features selection

DL features were merged with Radiomics features to form a combined DL-radiomics dataset for each image. Z-score normalization was applied to all extracted features, including Radiomics, DL, and combined DL-radiomics, for standardization. Regarding the fusion of DL-Radiomics features, we chose to perform Z-score normalization after the features have been merged. The Spearman correlation coefficient was used to assess feature correlations, retaining one feature from pairs with a correlation above 0.9. The least absolute shrinkage and selection operator (LASSO) logistic regression was then applied, with penalty parameter tuning via 10-fold cross-validation to identify CLN-status-related features with nonzero coefficients in the training cohort.

### Model development and assessment

Three feature sets (Radiomics, DL, and clinical) were fed into various kinds of machine learning algorithm in different combinations, resulting in seven models: Clinical, Radiomics, DL, Radiomics-Clinical, DL-Clinical, DL-Radiomics, and DL-Radiomics-Clinical (DRC). The performance of seven models was assessed using the area under the curve (AUC) of the receiver operating characteristics curve (ROC) with a 95% confidence interval (CI), Acc (accuracy), sensitivity and specificity for three cohorts.

After comparing the performance of each model, the internal (*n* = 61) and external (*n* = 111) testing cohorts were then introduced to assess the selected model’s stability. Nuclear medicine radiologist 1, 3, 4 (F.F.C, T.T.L, and J.J.Z) and four thoracic surgeons (Y.H.Y., F.C.B., J.M.X, and J.H.) were invited to outline the ROIs of the test cohort. Our goal is to test the stability of the model across different datasets, and to investigate the effect of the minor differences between the individual radiologists and specialist physicians in delineating the ROIs. The features extracted from the ROIs were used to test the selected model by comparing the AUC values in both internal and external test sets.

Additionally, we stratified patients with complete follow-up survival data into high-risk and low-risk groups based on the scores generated by the best model. The objective was to determine if there were any survival differences between patients grouped according to the selected model and those with or without CLNM.

### Follow-up and survival analysis

After the surgery (performed by Y.Q. and X.N.L.), patients were scheduled for visit at the 1st and 4th month. then regular visit every 6 months for the first 2 years after the surgery, and annually thereafter either at our outpatient’s clinic or by the physician around their residence. Data were gathered from outpatient records or phone calls until death or cancer recurrence. Routine chest-abdomen CT, esophagoscopy, and neck ultrasound were scheduled every 6 months in the first year, then annually. Other examinations like bone scan, PET/CT and bronchoscopy were performed based on clinical indications.

Follow-up was completed for 107 patients across both cohorts. Using the Youden index, the optimal DRC score cutoff divided patients into low-risk and high-risk groups to explore the correlation between DRCS and clinical outcomes. Our goal was to assess the DRC model’s prognostic value for these patients.

### Statistical analysis

Data analysis was conducted using Python (version 3.7.6) and R (version 3.6.3). The normality of continuous variables was assessed using the Kolmogorov-Smirnov test. The homogeneity of variance for continuous variables was evaluated with the Levene test. Inter-group differences were compared using the Mann-Whitney U test or Student’s t-test. Differences in categorical variables were compared using the Chi-squared test or Fisher’s exact test. A two-sided *p* < 0.05 indicated statistical significance. Univariable and multivariable logistic regressions identified CLNM clinical predictors in ESCC. Model diagnostic performance was assessed by ROC curve and AUC. Kaplan-Meier analysis evaluated prognostic differences, with the log-rank test comparing survival rates. Overall survival (OS) was defined as the duration from surgery to death due to any cause. Disease-free survival (DFS) was defined as the period from surgery to either recurrence, metastasis, or death from any cause. All statistical analyses were two-sided, with a P-value of less than 0.05 deemed to indicate a statistically significant difference.

## Results

### Clinical characteristics

Table 1 shows the clinical factors of the studied patients. After careful screening according to our inclusion criteria, 411 patients were finally included. There were no statistically significant differences in age, sex, tumor differentiation, T stage in three cohorts. RLNM and tumor location showed statistical differences only in the training cohort, vascular infiltration only in the external testing cohort, and tumor thickness in both. Tumor length, SLNM, No.100 ~ No.103 LNM, Thoracic or abdominal LNM differed statistically across all three cohorts. As shown in Supplementary Tables 1, to construct the clinical model, we conducted both univariable and multivariable logistic regression analyses on the training set and found tumor location to be an independent predictor for CLNM (*p* < 0.05). Survival analysis revealed the CLNM group (*n* = 32) had median OS and DFS of 22.5 and 16.5 months, respectively, versus 37 and 31 months for the non-CLNM group (*n* = 75). High-risk group (*n* = 29) had median OS and DFS of 15.0 and 14.1 months, significantly shorter than 38.2 and 35.0 months of the low-risk group (*n* = 78), respectively.

**Table 1:** Clinical and PET/CT morphological characteristics of patients in the training and testing cohorts

Characteristic	Training cohort				Internal testing cohort				External testing cohort				Survival test set				Survival test set		
	CLNM-positive	CLNM-negative	*p* value		CLNM-positive	CLNM-negative	*p* value		CLNM-positive	CLNM-negative	*p* value		CLNM-positive	CLNM-negative	*p* value		High-risk	Low-risk	*p* value
Age (years)			0.175				0.170				0.952				0.350				0.263
Mean ± SD	63.74 ± 8.10	65.34 ± 7.79			61.33 ± 8.87	66.80 ± 8.62			64.00 ± 7.67	63.68 ± 8.09			63.50 ± 7.82	65.16 ± 8.59			63.17 ± 7.94	65.22 ± 8.50	
Tumor thickness (mm)			0.035				0.941				0.033				0.874				0.891
Mean ± SD	26.54 ± 9.03	23.20 ± 11.42			23.87 ± 12.05	22.89 ± 9.53			18.81 ± 6.69	16.55 ± 11.13			19.76 ± 7.97	20.07 ± 10.01			19.83 ± 7.90	20.03 ± 9.96	
Tumor length (mm)			0.032				0.045				0.010				0.004				0.060
Mean ± SD	53.13 ± 24.21	43.47 ± 21.53			70.59 ± 30.73	44.21 ± 21.15			52.43 ± 31.26	36.31 ± 19.04			55.84 ± 31.50	40.72 ± 20.15			55.21 ± 32.38	41.54 ± 20.58	
Sex			0.701				1.000				0.354				0.528				0.718
Male	31 (81.58)	172 (85.57)			5 (83.33)	49 (89.09)			20 (76.92)	55 (64.71)			25 (78.12)	64 (85.33)			23 (79.31)	66 (84.62)	
Female	7 (18.42)	29 (14.43)			1 (16.67)	6 (10.91)			6 (23.08)	30 (35.29)			7 (21.88)	11 (14.67)			6 (20.69)	12 (15.38)	
Tumor differentiation			0.111				0.603				0.309				0.041				0.083
high differentiation	2 (5.26)	38 (18.91)			0 (0.00)	8 (14.55)			0 (0.00)	3 (3.53)			0 (0.00)	8 (10.67)			0 (0.00)	8 (10.26)	
poorly differentiated	15 (39.47)	63 (31.34)			2 (33.33)	15 (27.27)			2 (7.69)	14 (16.47)			4 (12.50)	18 (24.00)			4 (13.79)	18 (23.08)	
moderately differentiated	21 (55.26)	100 (49.75)			4 (66.67)	32 (58.18)			24 (92.31)	68 (80.00)			28 (87.50)	49 (65.33)			25 (86.21)	52 (66.67)	
Vascular infiltration			0.419				0.427				0.015				0.179				0.754
Yes	18 (47.37)	78 (38.81)			3 (50.00)	14 (25.45)			9 (34.62)	10 (11.76)			20 (62.50)	17 (22.67)			9 (31.03)	20 (25.64)	
No	20 (52.63)	123 (61.19)			3 (50.00)	41 (74.55)			17 (65.38)	75 (88.24)			12 (37.50)	58 (77.33)			20 (68.97)	58 (74.36)	
T stage			0.096				0.925				0.057				0.029				0.051
T1a	0 (0.00)	10 (4.98)			0 (0.00)	2 (3.64)			1 (3.85)	6 (7.06)			1 (3.12)	3 (4.00)			1 (3.45)	3 (3.85)	
T1b	2 (5.26)	39 (19.40)			2 (33.33)	11 (20.00)			1 (3.85)	19 (22.35)			3 (9.38)	14 (18.67)			3 (10.34)	14 (17.95)	
T2	7 (18.42)	34 (16.92)			1 (16.67)	13 (23.64)			20 (76.92)	38 (44.71)			21 (65.62)	24 (32.00)			19 (65.52)	26 (33.33)	
T3	29 (76.32)	116 (57.71)			3 (50.00)	28 (50.91)			4 (15.38)	21 (24.71)			7 (21.88)	32 (42.67)			6 (20.69)	33 (42.31)	
T4	0 (0.00)	2 (1.00)			0 (0.00)	1 (1.82)			0 (0.00)	1 (1.18)			0 (0.00)	2 (2.67)			0 (0.00)	2 (2.56)	
RLNM			0.027				0.059				0.498				1.000				1.000
Yes	16 (42.11)	47 (23.38)			4 (66.67)	12 (21.82)			2 (7.69)	2 (2.35)			6 (18.75)	13 (17.33)			5 (17.24)	14 (17.95)	
No	22 (57.89)	154 (76.62)			2 (33.33)	43 (78.18)			24 (92.31)	83 (97.65)			26 (81.25)	62 (82.67)			24 (82.76)	64 (82.05)	
SLNM			< 0.001				< 0.001				< 0.001				< 0.001				< 0.001
Yes	28 (73.68)	0 (0.00)			5 (83.33)	0 (0.00)			21 (80.77)	0 (0.00)			26 (81.25)	0 (0.00)			24 (82.76)	2 (2.56)	
No	10 (26.32)	201 (100.00)			1 (16.67)	55 (100.00)			5 (19.23)	85 (100.00)			6 (18.75)	75 (100.00)			5 (17.24)	76 (97.44)	
No.100 ~ No.103 LNM			< 0.001				< 0.001				< 0.001				< 0.001				< 0.001
Yes	13 (34.21)	0 (0.00)			1 (16.67)	0 (0.00)			13 (50.00)	0 (0.00)			14 (43.75)	0 (0.00)			12 (41.38)	2 (2.56)	
No	25 (65.79)	201 (100.00)			5 (83.33)	55 (100.00)			13 (50.00)	85 (100.00)			18 (56.25)	75 (100.00)			26 (68.42)	76 (97.44)	
Thoracic or abdominal LNM			0.032				0.043				< 0.001				< 0.001				< 0.001
Yes	21 (55.26)	71 (35.32)			4 (66.67)	11 (20.00)			14 (53.85)	14 (16.47)			18 (56.25)	16 (21.33)			17 (58.62)	17 (21.79)	
No	17 (44.74)	130 (64.68)			2 (33.33)	44 (80.00)			12 (46.15)	71 (83.53)			14 (43.75)	59 (78.67)			12 (41.38)	61 (78.21)	
Location			0.004				0.899				0.777				0.546				0.286
middle and low	23 (60.53)	166 (82.59)			4 (66.67)	43 (78.18)			16 (61.54)	57 (67.06)			20 (62.50)	53 (70.67)			17 (58.62)	56 (71.79)	
upper	15 (39.47)	35 (17.41)			2 (33.33)	12 (21.82)			10 (38.46)	28 (32.94)			12 (37.50)	22 (29.33)			12 (41.38)	22 (28.21)	

Abbreviations: CLNM, cervical lymph node metastasis; RLNM, recurrent laryngeal nerve lymph node metastasis; SLNM, supraclavicular lymph node metastasis

### Radiomics and DL features analysis

For each sample, we extracted 428 PET/CT-based Radiomics features from the GTV and CLN delineated layer by layer and 256 PET/CT-based DL features from the maximum cross-section of GTV and CLN images. Supplementary Fig. 1 showed the proportion of different kinds of features. Radiomics features includes 72 first-order, 96 Gray level co-occurrence matrix (GLCM), 64 Gray level size zone matrix (GLSZM), 56 Gray level dependence matrix (GLDM), 64 Gray level run length matrix (GLRLM), 20 Neighbouring Gray Tone Difference Matrix (NGTDM) and 56 shape features. The meanings of various kinds of Radiomics features can be found on https://pyradiomics.readthedocs.io/en/latest/features.html#radiomics-features-label. DL dimensions were reduced to 64 to enhance model generalization and mitigate overfitting risk. After LASSO regression, using 10-fold cross-validation to select λ, optimal λ values for Radiomics, DL, and DL-Radiomics models were 0.0193, 0.0018, and 0.0126 (Supplementary Fig. 2), respectively, selecting 11, 25, and 26 features for model construction (Supplementary Fig. 3). The description of the selected Radiomics features for DL-Radiomics model was demonstrated in Supplementary Text 3.

### Selection of the DL models and the development of different models

**Fig. 3:**
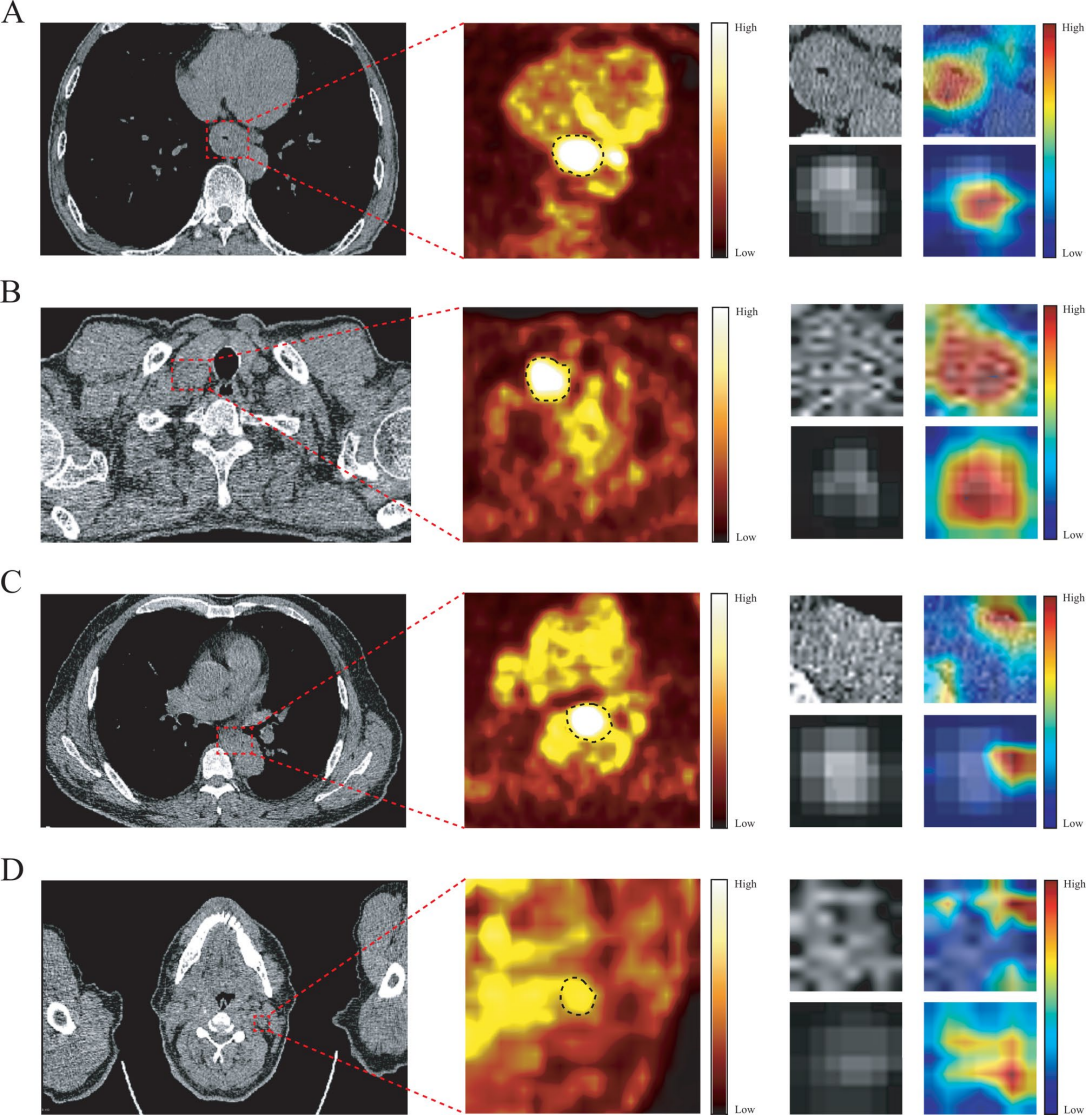
The attention regions of DL (resnet50) in a CLNM patient’s tumor **(A)** and CLN **(B)**, and a non-CLNM patient’s tumor **(C)** and CLN **(D)**; DL, deep learning; ESCC, esophageal squamous cell carcinoma; CLNM, cervical lymph node metastasis

Internal cohort patients were randomly divided into training and testing groups at an 8:2 ratio, with model optimal parameter based on the training group. By adjusting the hyperparameters of each DL model, Resnet50 was selected as the superior DL model which is outperformed other models (Supplementary Table 2) which used the optimizer of stochastic gradient descent, an initial learning rate of 0.001, and a batch size of 32. Gradient-weighted class activation mapping (Grad-CAM) was utilized to enhance DL model interpretability, highlighting how tumor and CLN areas influence CLNM detection, particularly identifying the heartland region as a key metastasis indicator (Fig. 3).

### Assessment of the performance of seven models

**Fig. 4:**
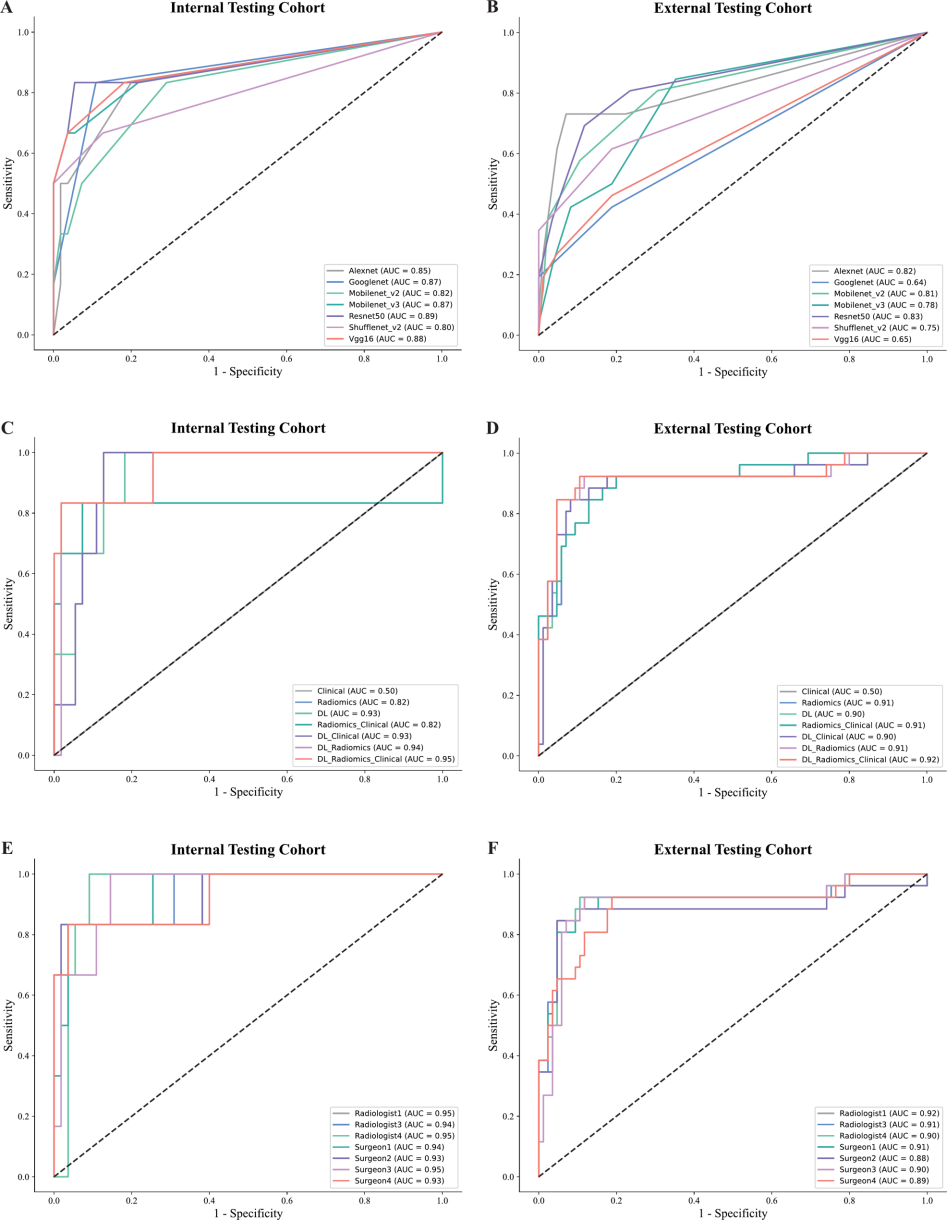
ROC curves of different deep learning models in the internal testing **(A)** and external testing **(B)** cohorts; ROC curves of Radiomics model, DL model and DL-Radiomics model in the internal testing **(C)** and external testing **(D)** cohorts; ROC curves of DL-Radiomics model using ROIs delineated by different physicians in the internal testing **(E)** and external testing **(F)** cohorts; ROC, receiver operating characteristic

Supplementary Table 3 showed the comparison results of seven machine learning algorithms. We found that LR (logistic regression) was the best classifier and chose it as the best machine learning model for accepting different feature inputs. In the training, internal and external testing cohorts, we comprehensively assessed the AUC, accuracy, sensitivity, and specificity of the seven models. The DL-Radiomics-Clinical (DRC) model exhibited higher AUC than the other six models in three cohorts (Table 2). In all three cohorts, the DRC model consistently demonstrated superior performance. After the differentiation of DRC mocel, the samples of the three cohorts were divided into high-risk group and low-risk group. The number of high-risk group in three cohorts was 39 (training cohort), 5 (internal testing cohort) and 32 (external testing cohort). The number of low-risk group in three cohorts was 200 (training cohort), 56 (internal testing cohort) and 79 (external testing cohort). In the training, internal and external testing cohorts, the DRC model achieved the highest AUC values among all models, with values of 0.999, 0.955 and 0.916, respectively. Figure 4 illustrated the ROC curves and AUC values for various DL models and seven models using different combinations of Radiomics, DL and clinical features. It also presented the ROC curves and AUC values of the DRC model under ROIs delineated by different physicians highlighting the model’s consistent effectiveness and adaptability. To enhance the interpretability of the model features, we used the GraphViz package (version 0.19.2) to output the coefficients of each feature in the DRC model, with the results recorded in Supplementary Text 4. Additionally, we also plotted a nomogram for the DRC model (Supplementary Fig. 4).

**Table 2:** Performance of different models for detecting CLNM in ESCC PET/CT images in three cohorts

Model	Cohort	Acc	AUC	95%CI	Sensitivity	Specificity
Clinical	Training	0.803	0.614	0.5107–0.7166	0.0000	1.0000
	Internal testing	0.902	0.500	0.2548–0.7452	0.0000	1.0000
	External testing	0.766	0.500	0.3726–0.6273	0.0000	1.0000
Radiomics	Training	0.941	0.965	0.9205-1.0000	0.7895	0.9801
	Internal testing	0.918	0.818	0.6057-1.0000	0.6667	0.9455
	External testing	0.856	0.909	0.8316–0.9882	0.4615	0.9765
DL	Training	0.987	0.999	0.9985-1.0000	0.8947	0.9950
	Internal testing	0.852	0.927	0.8588-1.0000	0.6667	0.8727
	External testing	0.874	0.904	0.8228–0.9843	0.5000	0.9529
Radiomics-Clinical	Training	0.941	0.966	0.9214-1.0000	0.7368	0.9900
	Internal testing	0.902	0.818	0.6057-1.0000	0.6667	1.0000
	External testing	0.865	0.911	0.8328–0.9888	0.4615	0.9882
DL-Clinical	Training	0.987	0.999	0.9985-1.0000	0.8421	0.9950
	Internal testing	0.869	0.930	0.7866-1.0000	0.6667	0.9455
	External testing	0.865	0.904	0.8247–0.9852	0.5385	0.9647
DL-Radiomics	Training	0.987	0.999	0.9988-1.0000	0.9211	0.9950
	Internal testing	0.934	0.942	0.8107-1.0000	0.8333	0.9818
	External testing	0.883	0.914	0.8379–0.9910	0.5769	0.9765
DL-Radiomics-Clinical	Training	0.987	0.999	0.9988-1.0000	0.9474	1.0000
	Internal testing	0.951	0.955	0.8696-1.0000	0.8333	1.0000
	External testing	0.892	0.916	0.8342–0.9975	0.6154	0.9765

Abbreviations: Acc, accuracy; AUC, area under curve; CI, confidence interval; DL, deep learning.

### Correlation of DRCS with prognosis of ESCC patients

**Fig. 5:**
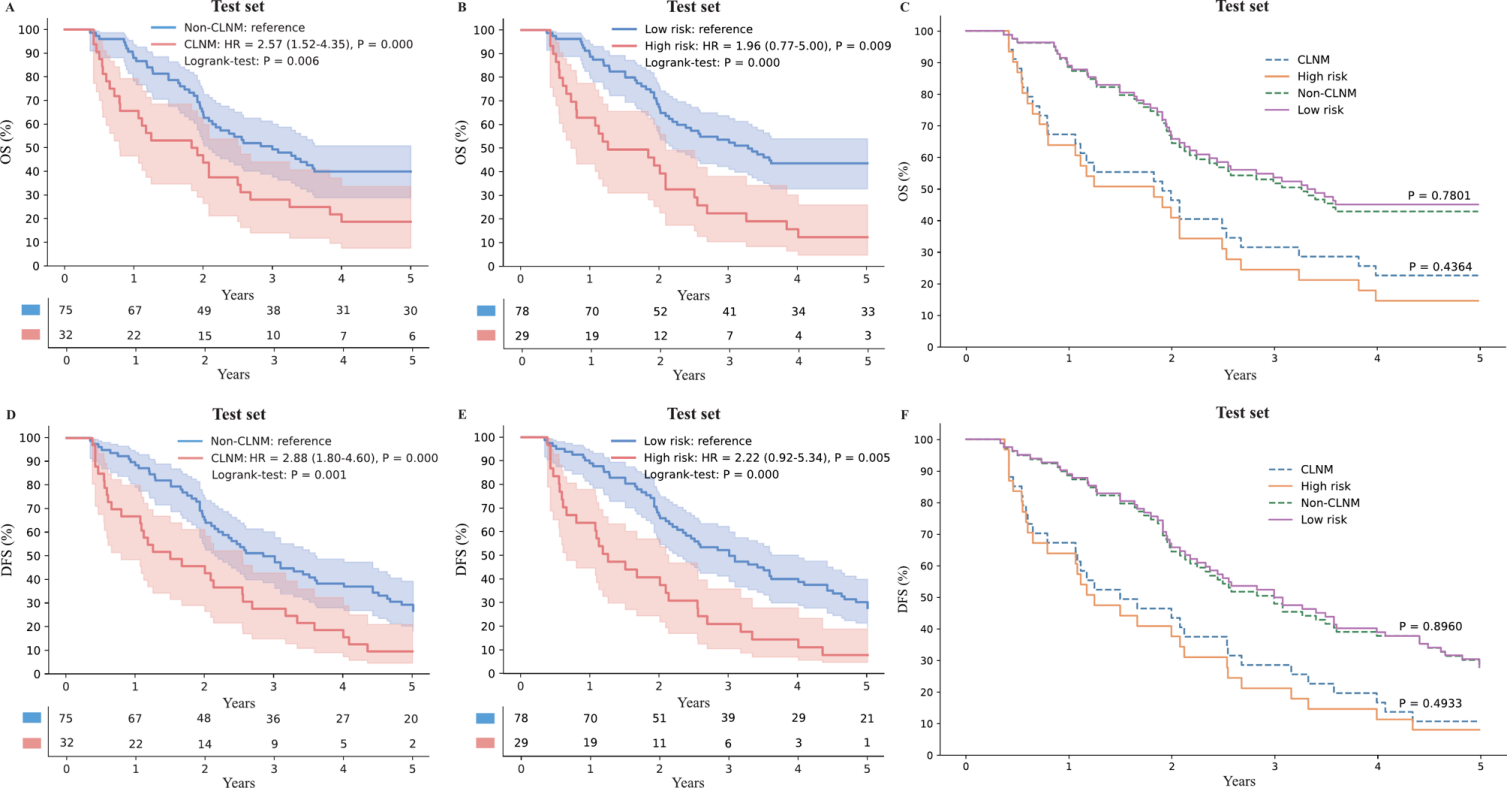
The Kaplan-Meier curves show overall survival and disease-free survival of CLNM and non-CLNM groups (**A**, **D**), high-risk and low-risk group (**B**, **E**), CLNM, non-CLNM, high-risk and low-risk group (**C**, **F**). OS, overall survival; DFS, disease-free survival; H, high-risk; L, low-risk

Among the 172 patients in the internal testing set (*n* = 61) and the external testing set (*n* = 111), we excluded 65 patients who were lost to 5-year follow-up and selected a total of 107 patients who had at least five years of complete follow-up survival data and included these 107 patients in the survival analysis. Figure 5 showed the Kaplan-Meier survival curve of different groups. Patients in the CLNM group had decreased OS (hazard ratio [HR], 2.57 [95% CI: 1.52, 4.35]; Logrank-test: *P* = 0.006) and DFS (HR, 2.88 [95% CI: 1.80, 4.60]; Logrank-test: *P* = 0.001) compared with patients in the non-CLNM group in the test set (Fig. 5A and D). Patients in the high-risk group had decreased OS (HR, 1.96 [95% CI: 0.77, 5.00]; Logrank-test: *P* < 0.001) and DFS (HR, 2.22 [95% CI: 0.92, 5.34]; Logrank-test: *P* < 0.001) compared with patients in the low-risk group in the test set (Fig. 5B and E). Logrank-test confirmed longer OS and DFS for non-CLNM over CLNM patients, and for low over high DRCS patients (*p* < 0.01). No significant OS or DFS differences were observed between CLNM and high-risk or non-CLNM and low-risk groups, suggesting CLNM prediction is closely related to patient prognosis.

### Subgroup analysis

A total of 172 patients from the internal (*n* = 61) and external (*n* = 111) testing cohorts were divided into three subgroups based on T stage: T0 ~ 1 group, T2 group, and T3 ~ 4 group. Supplementary Table 4 shows the clinical baseline data of these three subgroups. The AUC was 0.855 (T0 ~ 1group), 0.948 (T2 group) and 0.908 (T3 ~ 4 group) respectively which meant DRC model exhibited excellent performance across the three subgroups.

## Discussion

This study aimed to evaluate the efficacy of Radiomics, DL, and clinical factors for CLNM identification and develop a reliable prediction model using PET/CT images and clinical features. The DRC model utilizing the LR classifier demonstrated the highest performance among the seven models in both internal and external testing cohorts. More importantly, the reliability of this model also lies in its diversity-tolerant ability under ROI delineation heterogeneity by individual experience of image reading. The selected features of the DRC model overlapped with those of the Radiomics model (7 Radiomics features) and the DL model (15 DL features) which were showed in Supplementary Text 5. We speculated that the DRC model, when combining Radiomics features and DL features, eliminated some relatively unimportant features. This allowed the DRC model to exhibit superior performance compared to the single Radiomics or DL model.

Radiomics-based model for predicting CLNM in ESCC was rarely reported. Xie et al. developed a LNM predicating model using Radiomics features from CT images of lymph node, they reported an AUC of 0.891 in their cervical test set [42]. The DRC model demonstrated a better performance (AUC = 0.916) in our external testing cohort. Furthermore, the performance of our model was enhanced in the aspect of tolerant of human derived diversity during ROI delineation, which may attribute to comprehensive utilization of Radiomics, DL and clinical features. In addition, integrating Radiomics features from multi-regional image data were reported to improve prognosis prediction [43]. Thus, we would also consider extracting and analyzing DL/Radiomics features from both tumor and lymph node images as another asset of DRC model, which would likely enhance our DRC model’s robustness to some extent. Xie et al. [42] believed the Radiomics feature of GLSZM-GrayLevelNonUniformity from CT images of cervical lymph nodes could serve as an effective predictor for CLNM in ESCC which coincided with our results. However, building on their research, we supplemented PET/CT image features from the tumor region and PET image features from cervical lymph nodes, and combined Radiomics with DL which might be the reason we achieved better results.

We concerned that diverse tumor boundary delineations might affect our DRC model’s prediction accuracy. However, it performed well, even with ROIs delineated by thoracic surgeons with less radiology-based image reading experience. This may be attributed to the introduction of the models using deep learning approaches other than Radiomics alone, which was dependent on precise tumor boundary delineation and did not consider potential details that may be present in the peritumoral micro-environment. The result demonstrated the advanced deep learning approaches did not require accurate segmentation to achieve better performance in diagnosis and prediction [44], which in a way strongly indicated the DRC model’s general applicability for physicians that did not demand requisite professional background of nuclear medicine and radiology when drawing the ROIs.

Our model has potential value in guiding the extend of lymphadenectomy in ESCC patients who underwent esophagectomy. There has been a long-standing lack of consensus regarding the optimal extent of lymphadenectom*y* for ESCC patients. Wider dissection may lead to better staging, local control, and survival, notably in upper chest EC patients [45]. However, Li, B. et al. reported that 3-field lymphadenectomy didn’t improve OS or DFS for patients with middle and lower chest ESCC, even under the fact that 21.5% of patients were found to have unforeseen invaded CLN during the surgery [46]. Previous studies have reported that 3-field lymphadenectomy increases the incidence of postoperative complications [47]. In a retrospective analysis of Fujita et al., 122 out of 176 EC patients (69%) who underwent 3-field lymphadenectomy experienced postoperative recurrent laryngeal nerve palsy [48], which may predispose patients to severe complications like aspiration pneumonia and detrimentally impact their quality of life by aphonia paralytica [46, 49]. Therefore, prophylactic 3-field lymphadenectomy might be deemed excessive in patients without preoperative evidence of CLNM [49]. Non-invasive and accurate evaluation of CLNM by our DRC model might help to decide the extend of intraoperative lymphadenectomy, for reducing perioperative complications, ensuring post-surgery quality of life, not compromising oncological benefit from surgery.

Our study showed patients in the CLNM group had significantly lower OS and DFS compared to the non-CLNM group, consistent with prior research findings [6]. Furthermore, After DRC model reclassification, high-risk patients had significantly worse survival than low-risk ones, indicating accurate preoperative CLN prediction not only impacts lymphadenectomy decisions but provides prognostic insights. This information is crucial for physicians’ perioperative and postoperative treatment choices. We observed that within the DRC model’s high-risk group, some non-CLNM patients have poor prognoses and increased HR, indicating CLNM isn’t the only outcome factor. Prior research [50] has investigated the potential benefits of integrating ctDNA testing with PET/CT imaging. This method could improve recurrence risk prediction in post-treatment ESCC, allowing better minimal residual disease (MRD) estimation. It suggests PET/CT might reveal concealed MRD and prognostic information. We believe some patients may have poor outcomes from postoperative MRD, even without CLNM. Currently, the standard management for patients following neoadjuvant radiochemotherapy and surgery is monitoring. For patients with poor prognoses, adjuvant therapies are clearly needed to enhance outcomes. The CheckMate 577 trial, targeting resectable, locally advanced esophageal or gastroesophageal junction cancer, showed nivolumab as adjuvant therapy outperformed placebo, notably improving OS and DFS primary endpoints [51]. This implies that for patients classified as high-risk by the model, postoperative treatment may require not only monitoring but also the application of different Immune Checkpoint Inhibitors (ICIs) to improve their prognosis.

Our study showed that it was entirely possible to accurately predict CLNM of ESCC non-invasively before surgery. Even when using the ROIs delineated by surgeons, the DRC model still performed stably. The DRC model used LR as the final classifier, which meant we could extract and quantify the imaging features of each ESCC patient. Ultimately, a specific DRC score was output through the LR formula. The level of this score could assist surgeons in preoperatively formulating individualized surgical methods or lymph node dissection strategies. Although PET/CT scans have gradually become popular in large hospitals, the high cost will still be a challenge for the clinical application of the DRC model in the future. As can be seen from the comparative studies mentioned above, the advantages of PET/CT are irreplaceable by CT. Three-field lymph node dissection, including cervical lymph node dissection, often represents a more challenging and aggressive method with a higher incidence of postoperative complications. However, if cervical lymph node dissection is neglected, the potentially missed malignant cervical lymph nodes could have a devastating impact on the prognosis of ESCC patients. Therefore, accurately predicting CLNM is extremely necessary which makes us believe to improve the prediction probability of CLNM through imaging models, PET/CT is indispensable.

Several limitations should be addressed. First, the infrequent use of PET/CT scans and its retrospective design could lead to selection bias from a small sample size, necessitating larger cohorts and prospective trials for clinical application. Second, exclusively involving Asian ESCC patients, the model’s applicability to other ethnicities or to esophageal adenocarcinoma patients is uncertain, highlighting the need for models based on diverse patient groups. The samples being exclusively from China may limit the generalizability of the results to other populations or ethnic groups. The lack of diversity in the sample could introduce selection bias. Third, when performing cervical lymph node dissection, the coverage although including the clearance of No. 104 and No. 101 lymph nodes, might not entirely encompass the cervical region. However, we believe that in the process of model training, focusing on the delineation of specific CLNs with corresponding pathological results could enable the DRC model to maintain strong generalizability and performance when predicting CLNs. Finally, we solely utilized PET/CT imaging in model construction. Our future work will include investigating magnetic resonance imaging and pathological images of patients, focusing on multi-omics experiments. We will attempt to conduct international multicenter studies, including patients from different countries or races, to reduce selection bias caused by different populations or ethnic groups.

## Conclusions

In conclusion, our study demonstrates the potential of AI in revealing concealed information in PET/CT images, and the DRC model exhibits outstanding performance. The developed models hold the potential to identify ESCC patients without CLNM, enabling them to avoid unnecessary lymph node dissection.

## Electronic supplementary material

Below is the link to the electronic supplementary material.

Supplementary Material 1

Supplementary Material 2

Supplementary Material 3

## Data Availability

No datasets were generated or analysed during the current study.

## Abbreviations

^18^F-FDG: ^18^F-fluorodeoxyglucose
CLNM: Cervical lymph node metastasis
ESCC: Esophageal squamous cell carcinoma
DRC: DL-Radiomics-Clinical
DL: Deep learning
CNNs: Convolutional neural networks
RLNM: Recurrent laryngeal nerve lymph node metastasis
SLNM: Supraclavicular lymph node metastasis
ROIs: Regions of interest
ICCs: Intraclass correlation coefficients
LASSO: Least absolute shrinkage and selection operator
LR: Logistic regression
ROC: Receiver operating characteristics curve
OS: Overall survival
DFS: Disease-free survival
Grad-CAM: Gradient-weighted class activation mapping
MRD: Minimal residual disease
