# Supplementary material for: A 18F-FDG PET/CT-based deep learning-radiomics-clinical model for prediction of cervical lymph node metastasis in esophageal squamous cell carcinoma
Source: Cancer Imaging. 2024 Nov 12;24:153. doi: 10.1186/s40644-024-00799-0 (PMC11556142; doi:10.1186/s40644-024-00799-0)
Supplement: Supplementary file 1 — Supplementary Material 1 [file 40644_2024_799_MOESM1_ESM.docx]

**SUPPLEMENTARY TABLE**

**TITLE: A ^18^F-FDG PET/CT-based deep learning-radiomics-clinical model for prediction of cervical lymph node metastasis in esophageal squamous cell carcinoma**

**CONTENT**

1. **Supplementary Table 1:** Univariable and multivariable logistic regression analysis of factors in the training cohort.
2. **Supplementary Table 2:** Comparison results of performance of different DL models.
3. **Supplementary Table 3:** Performance of seven kinds of machine learning algorithm using different features for detecting CLNM in ESCC PET/CT images in three cohorts
4. **Supplementary Table 4:** Subgroup analysis based on T stage

| **Supplementary Table 1** Univariable and multivariable logistic regression analysis of factors in the training cohort | | | | | | |
| --- | --- | --- | --- | --- | --- | --- |
| Factors | Univariable logistic regression | | | Multivariable logistic regression | | |
|  | OR (95%CI) | *p* value |  | OR (95%CI) | *p* value |  |
| Tumor differentiation | 1.047 (0.994-1.103) | 0.149 |  |  |  |  |
| Vascular infiltration | 1.049 (0.969-1.135) | 0.326 |  |  |  |  |
| Sex | 0.959 (0.860-1.070) | 0.530 |  |  |  |  |
| Tumor thickness | 1.004 (1.000-1.007) | 0.089 |  |  |  |  |
| Tumor length | 1.003 (1.001-1.004) | 0.014 |  | 1.002 (1.000-1.004) | 0.113 |  |
| Age | 0.997 (0.992-1.001) | 0.248 |  |  |  |  |
| Tstage | 1.067 (1.022-1.113) | 0.012 |  | 1.033 (0.986-1.082) | 0.249 |  |
| RLNM | 1.138 (1.042-1.242) | 0.016 |  | 1.098 (1.006-1.198) | 0.080 |  |
| Tumor location | 0.837 (0.761-0.919) | 0.002 |  | 0.861 (0.783-0.947) | 0.010 |  |
| OR, odds ratio; CI, confidence interval; RLNM, recurrent laryngeal nerve lymph node metastasis | | | | | | |

| **Supplementary Table 2** Comparison results of performance of different DL models | | | | | | |
| --- | --- | --- | --- | --- | --- | --- |
| Model | Cohort | Acc | AUC | 95%CI | Sensitivity | Specificity |
|  |  |  |  |  |  |  |
| Resnet50 | Training | 0.941 | 0.985 | 0.9737-0.9967 | 0.658 | 0.995 |
|  | Internal testing | 0.934 | 0.888 | 0.9617-1.0000 | 0.667 | 0.964 |
|  | External testing | 0.829 | 0.834 | 0.7396-0.9282 | 0.385 | 0.965 |
| Alexnet | Training | 0.925 | 0.958 | 0.9356-0.9802 | 0.579 | 0.990 |
|  | Internal testing | 0.918 | 0.853 | 0.6667-1.0000 | 0.500 | 0.964 |
|  | External testing | 0.874 | 0.817 | 0.7120-0.9224 | 0.615 | 0.953 |
| Googlenet | Training | 0.895 | 0.981 | 0.9702-0.9921 | 0.342 | 1.000 |
|  | Internal testing | 0.902 | 0.871 | 0.6999-1.0000 | 0.000 | 1.000 |
|  | External testing | 0.766 | 0.636 | 0.5245-0.7466 | 0.000 | 1.000 |
| Mobilenet_V2 | Training | 0.950 | 0.969 | 0.9486-0.9885 | 0.684 | 1.000 |
|  | Internal testing | 0.902 | 0.821 | 0.6221-1.0000 | 0.333 | 0.964 |
|  | External testing | 0.838 | 0.810 | 0.7101-0.9093 | 0.385 | 0.976 |
| Mobilenet_V3 | Training | 0.937 | 0.967 | 0.9462-0.9882 | 0.763 | 0.970 |
|  | Internal testing | 0.934 | 0.873 | 0.6777-1.0000 | 0.667 | 0.964 |
|  | External testing | 0.802 | 0.777 | 0.6786-0.8757 | 0.423 | 0.918 |
| Shufflenet_V2 | Training | 0.912 | 0.974 | 0.9592-0.9886 | 0.447 | 1.000 |
|  | Internal testing | 0.951 | 0.802 | 0.5733-1.0000 | 0.500 | 1.000 |
|  | External testing | 0.793 | 0.746 | 0.6363-0.8560 | 0.115 | 1.000 |
| Vgg16 | Training | 0.908 | 0.968 | 0.9501-0.9851 | 0.447 | 0.995 |
|  | Internal testing | 0.951 | 0.880 | 0.6912-1.0000 | 0.500 | 1.000 |
|  | External testing | 0.802 | 0.654 | 0.5425-0.7657 | 0.192 | 0.988 |
| Acc, accuracy; AUC, area under the curve; CI, confidence interval; PPV, positive predictive value; NPV, negative predictive value; DL, deep learning | | | | | | |

| **Supplementary Table 3** Performance of seven kinds of machine learning algorithm using different features for detecting CLNM in ESCC PET/CT images in three cohorts | | | | | | | | | | | | | | | |
| --- | --- | --- | --- | --- | --- | --- | --- | --- | --- | --- | --- | --- | --- | --- | --- |
|  |  | KNN | | SVM | | LR | | XGBoost | | RandomForest | | LightGBM | | MLP | |
| Model | Cohort | Acc | AUC (95%CI) | Acc | AUC (95%CI) | Acc | AUC (95%CI) | Acc | AUC (95%CI) | Acc | AUC (95%CI) | Acc | AUC (95%CI) | Acc | AUC (95%CI) |
|  |  |  |  |  |  |  |  |  |  |  |  |  |  |  |  |
| Clinical | Training | 0.841 | 0.610 (0.5273-0.6933) | 0.711 | 0.645 (0.5404-0.7487) | 0.803 | 0.614 (0.5107-0.7166) | 0.753 | 0.803 (0.7289-0.8768) | 0.741 | 0.871 (0.8192-0.9231) | 0.707 | 0.739 (0.6556-0.8224) | 0.690 | 0.560 (0.4576-0.6626) |
|  | Internal testing | 0.902 | 0.558 (0.3438-0.7714) | 0.639 | 0.630 (0.3542-0.9064) | 0.902 | 0.500 (0.2548-0.7452) | 0.852 | 0.585 (0.2438-0.9259) | 0.508 | 0.548 (0.2884-0.8086) | 0.885 | 0.618 (0.3419-0.8945) | 0.705 | 0.673 (0.4187-0.9267) |
|  | External testing | 0.766 | 0.528 (0.4198-0.6354) | 0.694 | 0.503 (0.3648-0.6416) | 0.766 | 0.500 (0.3726-0.6273) | 0.595 | 0.472 (0.3498-0.5950) | 0.468 | 0.498 (0.3771-0.6179) | 0.730 | 0.557 (0.4190-0.6945) | 0.757 | 0.506 (0.3779-0.6339) |
| Radiomics | Training | 0.937 | 0.978 (0.9631-0.9928) | 0.954 | 0.957 (0.9156-0.9983) | 0.941 | 0.965 (0.9205-1.0000) | 0.967 | 0.994 (0.9853-1.0000) | 0.946 | 0.979 (0.9591-0.9996) | 0.916 | 0.971 (0.9495-0.9925) | 0.946 | 0.955 (0.9106-0.9993) |
|  | Internal testing | 0.934 | 0.888 (0.7050-1.0000) | 0.934 | 0.894 (0.6984-1.0000) | 0.918 | 0.818 (0.6057-1.0000) | 0.934 | 0.915 (0.7850-1.0000) | 0.934 | 0.948 (0.8898-1.0000) | 0.918 | 0.898 (0.7761-1.0000) | 0.951 | 0.927 (0.7961-1.0000) |
|  | External testing | 0.865 | 0.827 (0.7259-0.9284) | 0.883 | 0.842 (0.7986-1.0000) | 0.856 | 0.909 (0.8316-0.9882) | 0.883 | 0.902 (0.7530-1.0000) | 0.856 | 0.870 (0.7791-0.9607) | 0.883 | 0.828 (0.7484-1.0000) | 0.829 | 0.906 (0.8879-1.0000) |
| DL | Training | 0.941 | 0.985 (0.9737-0.9967) | 0.950 | 0.996 (0.9907-1.0000) | 0.987 | 0.999 (0.9985-1.0000) | 0.996 | 1.000 (1.0000-1.0000) | 0.950 | 0.993 (0.9866-1.0000) | 0.958 | 0.992 (0.9845-1.0000) | 0.975 | 0.997 (0.9924-1.0000) |
|  | Internal testing | 0.934 | 0.888 (0.6917-1.0000) | 0.787 | 0.948 (0.8741-1.0000) | 0.852 | 0.927 (0.8588-1.0000) | 0.738 | 0.909 (0.7896-1.0000) | 0.918 | 0.942 (0.8569-1.0000) | 0.902 | 0.902 (0.8509-1.0000) | 0.918 | 0.946 (0.9079-1.0000) |
|  | External testing | 0.829 | 0.834 (0.7396-0.9282) | 0.829 | 0.892 (0.8200-0.9637) | 0.874 | 0.904 (0.8228-0.9843) | 0.883 | 0.874 (0.7839-0.9640) | 0.757 | 0.839 (0.7448-0.9335) | 0.811 | 0.873 (0.7893-0.9564) | 0.838 | 0.881 (0.7987-0.9633) |
| Radiomics-Clinical | Training | 0.941 | 0.982 (0.9685-0.9951) | 0.954 | 0.958 (0.9174-0.9988) | 0.941 | 0.966 (0.9214-1.0000) | 0.958 | 0.996 (0.9912-1.0000) | 0.967 | 0.983 (0.9625-1.0000) | 0.895 | 0.973 (0.9527-0.9931) | 0.946 | 0.927 (0.7961-1.0000) |
|  | Internal testing | 0.918 | 0.871 (0.6592-1.0000) | 0.934 | 0.885 (0.6718-1.0000) | 0.902 | 0.818 (0.6057-1.0000) | 0.934 | 0.903 (0.7443-1.0000) | 0.918 | 0.945 (0.8604-1.0000) | 0.902 | 0.874 (0.7172-1.0000) | 0.951 | 0.918 (0.7695-1.0000) |
|  | External testing | 0.883 | 0.856 (0.7603-0.9511) | 0.883 | 0.839 (0.7733-1.0000) | 0.865 | 0.911 (0.8328-0.9888) | 0.883 | 0.905 (0.7636-1.0000) | 0.856 | 0.868 (0.7743-0.9614) | 0.883 | 0.836 (0.7669-1.0000) | 0.829 | 0.906 (0.8879-1.0000) |
| DL-Clinical | Training | 0.954 | 0.986 (0.9744-0.9974) | 0.950 | 0.996 (0.9907-1.0000) | 0.987 | 0.999 (0.9985-1.0000) | 0.996 | 1.000 (1.0000-1.0000) | 0.958 | 0.993 (0.9860-1.0000) | 0.954 | 0.992 (0.9834-1.0000) | 0.975 | 0.997 (0.9924-1.0000) |
|  | Internal testing | 0.934 | 0.874 (0.6514-1.0000) | 0.787 | 0.952 (0.8775-1.0000) | 0.869 | 0.930 (0.7866-1.0000) | 0.689 | 0.894 (0.7562-1.0000) | 0.918 | 0.945 (0.8604-1.0000) | 0.902 | 0.936 (0.8336-1.0000) | 0.918 | 0.946 (0.9079-1.0000) |
|  | External testing | 0.838 | 0.840 (0.7445-0.9356) | 0.829 | 0.891 (0.8179-0.9640) | 0.865 | 0.904 (0.8247-0.9852) | 0.874 | 0.868 (0.7735-0.9618) | 0.838 | 0.836 (0.7395-0.9320) | 0.847 | 0.875 (0.7937-0.9557) | 0.838 | 0.880 (0.7971-0.9631) |
| DL-Radiomics | Training | 0.958 | 0.988 (0.9782-0.9974) | 0.979 | 0.998 (0.9956-1.0000) | 0.987 | 0.999 (0.9988-1.0000) | 0.996 | 1.000 (1.0000-1.0000) | 0.958 | 0.996 (0.9923-1.0000) | 0.950 | 0.991 (0.9815-0.9999) | 0.958 | 0.997 (0.9937-1.0000) |
|  | Internal testing | 0.918 | 0.874 (0.6892-1.0593) | 0.934 | 0.952 (0.8666-1.0000) | 0.934 | 0.942 (0.8107-1.0000) | 0.869 | 0.902 (0.7444-1.0000) | 0.934 | 0.900 (0.7228-1.0000) | 0.951 | 0.933 (0.8138-1.0000) | 0.869 | 0.953 (0.9272-1.0000) |
|  | External testing | 0.829 | 0.892 (0.8066-0.9767) | 0.892 | 0.908 (0.8200-0.9811) | 0.883 | 0.914 (0.8379-0.9910) | 0.865 | 0.873 (0.7821-0.9632) | 0.892 | 0.909 (0.8189-0.9982) | 0.892 | 0.908 (0.8261-0.9897) | 0.856 | 0.906 (0.8379-0.9739) |
| DL-Radiomics-Clinical | Training | 0.954 | 0.990 (0.9812-0.9985) | 0.979 | 0.998 (0.9956-1.0000) | 0.987 | 0.999 (0.9988-1.0000) | 0.996 | 1.000 (1.0000-1.0000) | 0.958 | 0.996 (0.9920-1.0000) | 0.950 | 0.991 (0.9824-0.9999) | 0.958 | 0.997 (0.9937-1.0000) |
|  | Internal testing | 0.967 | 0.889 (0.7138-1.0000) | 0.934 | 0.952 (0.8666-1.0000) | 0.951 | 0.955 (0.8696-1.0000) | 0.996 | 0.898 (0.7357-1.0000) | 0.934 | 0.900 (0.7228-1.0000) | 0.951 | 0.933 (0.8075-1.0000) | 0.869 | 0.953 (0.9272-1.0000) |
|  | External testing | 0.829 | 0.896 (0.8130-0.9798) | 0.892 | 0.910 (0.8293-0.9851) | 0.892 | 0.916 (0.8342-0.9975) | 0.829 | 0.851 (0.7437-0.9590) | 0.892 | 0.912 (0.8268-0.9966) | 0.892 | 0.913 (0.8333-0.9920) | 0.856 | 0.905 (0.8371-0.9737) |
| Acc, accuracy; AUC, area under curve; CI, confidence interval; DL, deep learning; KNN, K-Nearest Neighbor; LR, Logistic Regression; SVM, Support Vector Machine; XGBoost, Extreme Gradient Boosting; LightGBM, Light Gradient Boosting Machine; MLP, Multilayer Perceptron | | | | | | | | | | | | | | | |

| **Supplementary Table 4** Subgroup analysis based on T stage | | | | | | | | | | | |
| --- | --- | --- | --- | --- | --- | --- | --- | --- | --- | --- | --- |
| Characteristic | T0~1 group | | |  | T2 group | | |  | T3~4 group | | |
|  | CLNM-positive | CLNM-negative | *p* value |  | CLNM-positive | CLNM-negative | *p* value |  | CLNM-positive | CLNM-negative | *p* value |
| Age (years) |  |  | 0.205 |  |  |  | 0.518 |  |  |  | 0.570 |
| Mean ± SD | 57.00 ± 5.10 | 62.08 ± 7.65 |  |  | 64.57 ± 8.52 | 65.98 ± 8.31 |  |  | 64.00 ± 5.35 | 65.94 ± 8.71 |  |
| Tumor thickness (mm) |  |  | 0.808 |  |  |  | 0.185 |  |  |  | 0.728 |
| Mean ± SD | 11.70 ± 5.42 | 12.62 ± 6.43 |  |  | 20.14 ± 6.30 | 18.72 ± 12.70 |  |  | 23.20 ± 11.14 | 24.13 ± 9.11 |  |
| Tumor length (mm) |  |  | 0.847 |  |  |  | 0.007 |  |  |  | 0.099 |
| Mean ± SD | 26.65 ± 11.45 | 28.52 ± 19.19 |  |  | 57.67 ± 32.66 | 37.98 ± 17.28 |  |  | 67.02 ± 27.90 | 48.96 ± 19.42 |  |
| Sex |  |  | 1.000 |  |  |  | 1.000 |  |  |  | 0.302 |
| Male | 3 (75.00) | 29 (76.32) |  |  | 15 (71.43) | 37 (72.55) |  |  | 7 (100.00) | 38 (74.51) |  |
| Female | 1 (25.00) | 9 (23.68) |  |  | 6 (28.57) | 14 (27.45) |  |  | 0 (0.00) | 13 (25.49) |  |
| Tumor differentiation |  |  | 0.064 |  |  |  | 0.069 |  |  |  | 0.515 |
| high differentiation | 0 (0.00) | 3 (7.89) |  |  | 0 (0.00) | 5 (9.80) |  |  | 0 (0.00) | 3 (5.88) |  |
| poorly differentiated | 3 (75.00) | 8 (21.05) |  |  | 0 (0.00) | 6 (11.76) |  |  | 1 (14.29) | 15 (29.41) |  |
| moderately differentiated | 1 (25.00) | 27 (71.05) |  |  | 21 (100.00) | 40 (78.43) |  |  | 6 (85.71) | 33 (64.71) |  |
| Vascular infiltration |  |  | 0.969 |  |  |  | 0.052 |  |  |  | 1.000 |
| Yes | 1 (25.00) | 4 (10.53) |  |  | 9 (42.86) | 9 (17.65) |  |  | 2 (28.57) | 11 (21.57) |  |
| No | 3 (75.00) | 34 (89.47) |  |  | 12 (57.14) | 42 (82.35) |  |  | 5 (71.43) | 40 (78.43) |  |
| RLNM |  |  | 0.001 |  |  |  | 0.815 |  |  |  | 0.295 |
| Yes | 2 (50.00) | 0 (0.00) |  |  | 1 (4.76) | 5 (9.80) |  |  | 3 (42.86) | 9 (17.65) |  |
| No | 2 (50.00) | 38 (100.00) |  |  | 20 (95.24) | 46 (90.20) |  |  | 4 (57.14) | 42 (82.35) |  |
| SLNM |  |  |  |  |  |  | <0.001 |  |  |  | <0.001 |
| Yes | 3 (75.00) | 0 (0.00) |  |  | 16 (76.19) | 51 (100.00) |  |  | 7 (100.00) | 0 (0.00) |  |
| No | 1 (25.00) | 38 (100.00) |  |  | 5 (23.81) | 0 (0.00) |  |  | 0 (0.00) | 51 (100.00) |  |
| No.100~No.103 LNM |  |  | 0.163 |  |  |  | <0.001 |  |  |  | 1.000 |
| Yes | 1 (25.00) | 0 (0.00) |  |  | 0 (0.00) | 13 (61.90) |  |  | 0 (0.00) | 0 (0.00) |  |
| No | 3 (75.00) | 38 (100.00) |  |  | 51 (100.00) | 8 (38.10) |  |  | 7 (100.00) | 51 (100.00) |  |
| Thoracic or abdominal LNM |  |  | 0.097 |  |  |  | 0.013 |  |  |  | 1.000 |
| Yes | 2 (50.00) | 3 (7.89) |  |  | 12 (23.53) | 12 (57.14) |  |  | 2 (28.57) | 10 (19.61) |  |
| No | 2 (50.00) | 35 (92.11) |  |  | 39 (76.47) | 9 (42.86) |  |  | 5 (71.43) | 41 (80.39) |  |
| Location |  |  | 1.000 |  |  |  | 0.783 |  |  |  | 0.775 |
| middle and low | 29 (76.32) | 3 (75.00) |  |  | 35 (68.63) | 13 (61.90) |  |  | 4 (57.14) | 36 (70.59) |  |
| upper | 9 (23.68) | 1 (25.00) |  |  | 16 (31.37) | 8 (38.10) |  |  | 3 (42.86) | 15 (29.41) |  |
| DRC model's results |  |  |  |  |  |  |  |  |  |  |  |
| AUC (95% CI) | 0.855 (0.6151-1.0000) | | |  | 0.948 (0.8793-1.0000) | | |  | 0.908 (0.756-1.000) | | |
| Acc | 0.952 | | |  | 0.861 | | |  | 0.948 | | |
| Sensitivity | 0.750 | | |  | 0.619 | | |  | 0.714 | | |
| Specificity | 0.974 | | |  | 0.961 | | |  | 0.98 | | |
| CLNM, cervical lymph node metastasis; RLNM, recurrent laryngeal nerve lymph node metastasis; SLNM, supraclavicular lymph node metastasis; CI, confidence interval; Acc, accuracy; AUC, area under the curve | | | | | | | | | | | |
