# Supplementary material for: A 18F-FDG PET/CT-based deep learning-radiomics-clinical model for prediction of cervical lymph node metastasis in esophageal squamous cell carcinoma
Source: Cancer Imaging. 2024 Nov 12;24:153. doi: 10.1186/s40644-024-00799-0 (PMC11556142; doi:10.1186/s40644-024-00799-0)
Supplement: Supplementary file 2 — Supplementary Material 2 [file 40644_2024_799_MOESM2_ESM.docx]

**SUPPLEMENTARY TEXT**

**TITLE: A ^18^F-FDG PET/CT-based deep learning-radiomics-clinical model for prediction of cervical lymph node metastasis in esophageal squamous cell carcinoma**

**CONTENT**

1. **Supplementary Text 1: The parameters used for Radiomics features extraction from CT images using Pyradiomics**

For CT images, the imageType was set to LoG with sigma values of [1.0, 2.0, 3.0]. The normalizeScale was set to 1000 to ensure consistent gray value scaling. The interpolator used was 'sitkNearestNeighbor', and the resampledPixelSpacing was set to [1, 1, 1]. A padDistance of 10 was applied to accommodate large sigma values in the LoG filter. The correctMask option was enabled to ensure accurate mask alignment. The binWidth for image discretization was set to 5, and a voxelArrayShift of 1000 was applied to prevent negative values from being squared.

1. **Supplementary Text 2: The parameters used for Radiomics features extraction from PET images using Pyradiomics**

For PET images, the imageType was set to LoG with sigma values of [1.0, 2.0, 3.0, 4.0, 5.0]. The interpolator used was 'sitkNearestNeighbor', and the resampledPixelSpacing was set to [1, 1, 1]. The correctMask option was enabled to ensure accurate mask alignment, and the binWidth for image discretization was set to 0.25.

1. **Supplementary Text 3: The meaning of selected Radiomics features for DL-Radiomics model**

**(CLN_CT) glszm Gray Level Non-Uniformity Normalized:** It measures the variability of gray-level intensity values in the CT images of CLN, with a lower value indicating a greater similarity in intensity values.

**(CLN_PET) firstorder Variance:** It is the mean of the squared distances of each intensity value from the mean value of the first order features from the PET images of CLN. This is a measure of the spread of the distribution about the mean.

**(CLN_PET) gldm Dependence Non-Uniformity Normalized:** It measures the similarity of dependence throughout the PET images of CLN, with a lower value indicating more homogeneity among dependencies in the PET images of CLN.

**(CLN_PET) glszm Small Area Low Gray Level Emphasis:** It measures the proportion of the joint distribution of smaller size zones with lower gray-level values in the PET images of CLN.

**(Tumor CT) glrlm Long Run High Gray Level Emphasis:** It measures the joint distribution of shorter run lengths with higher gray-level values of CT images of tumor.

**(Tumor CT) glszm Zone Entropy:** It measures the uncertainty/randomness in the distribution of zone sizes and gray levels in the CT images of tumor. A higher value indicates more heterogeneneity in the texture patterns.

**(Tumor CT) shape Minor Axis Length:** This feature yield the second-largest axis length of the ROI-enclosing ellipsoid.

**(Tumor PET) glcm Imc2:** IMC2 features quantify the complexity of the texture by evaluating the mutual information between two probability distributions. A higher value indicates a stronger correlation between the two distributions and more complex textures. This feature is significant in image analysis and pattern recognition, helping to describe and distinguish different texture characteristics.

**(Tumor PET) glszm Large Area Low Gray Level Emphasis:** It measures the proportion of the joint distribution of larger size zones with lower gray-level values in the PET images of tumor.

**(Tumor PET) shape Maximum 3D Diameter:** Maximum 3D diameter is defined as the largest pairwise Euclidean distance between tumor surface mesh vertices.

1. **Supplementary Text 4: The establishment formula of DRC model**

**DL Radiomics Signature** = -4.543419851652009 +0.304276*CLN_CT_glszm_GrayLevelNonUniformityNormalized +0.994290*CLN_PET_firstorder_Variance +0.366004*CLN_PET_gldm_DependenceNonUniformityNormalized +0.542849*CLN_PET_glszm_SmallAreaLowGrayLevelEmphasis -0.025247*Tumor_CT_glrlm_LongRunHighGrayLevelEmphasis +0.405610*Tumor_CT_glszm_ZoneEntropy +1.178375*Tumor_CT_shape_MinorAxisLength -0.475411*Tumor_PET_glcm_Imc2 -0.772447*Tumor_PET_glszm_LargeAreaLowGrayLevelEmphasis +0.201373*Tumor_PET_shape_Maximum3DDiameter

+0.553382*CLN_CT_DL_0 +0.837018 * CLN_CT_DL_9 +0.045486*CLN_CT_DL_61 -1.590469*CLN_PET_DL_0 +0.806953*CLN_PET_DL_1 -0.441450*CLN_PET_DL_2 -1.110598*CLN_PET_DL_5 +0.306063*CLN_PET_DL_9 -0.077484*Tumor_CT_DL_3 -0.154887*Tumor_CT_DL_4 +0.511942*Tumor_CT_DL_11 +0.559111*Tumor_CT_DL_41 -0.597591*Tumor_CT_DL_57 +0.636833*Tumor_PET_DL_33 -0.530340*Tumor_PET_DL_34

**Clinical Signature** = 0.2803 -0.1534*Location

**DRC Model Score** = 0.0586 + 0.3165***DL Radiomics Signature** + 0.0049***Clinical Signature**

1. **Supplementary Text 5: The repeated features between DRC model and Radiomics model or DL model.**

**Radiomics features: (CLN_CT) glszm Gray Level Non-Uniformity Normalized:** It measures the variability of gray-level intensity values in the CT images of CLN, with a lower value indicating a greater similarity in intensity values.

**(CLN_PET) firstorder Variance:** It is the mean of the squared distances of each intensity value from the mean value of the first order features from the PET images of CLN. This is a measure of the spread of the distribution about the mean.

**(CLN_PET) gldm Dependence Non-Uniformity Normalized:** It measures the similarity of dependence throughout the PET images of CLN, with a lower value indicating more homogeneity among dependencies in the PET images of CLN.

**(CLN_PET) glszm Small Area Low Gray Level Emphasis:** It measures the proportion of the joint distribution of smaller size zones with lower gray-level values in the PET images of CLN.

**(Tumor CT) glrlm Long Run High Gray Level Emphasis:** It measures the joint distribution of shorter run lengths with higher gray-level values of CT images of tumor.

**(Tumor CT) glszm Zone Entropy:** It measures the uncertainty/randomness in the distribution of zone sizes and gray levels in the CT images of tumor. A higher value indicates more heterogeneneity in the texture patterns.

**(Tumor CT) shape Minor Axis Length:** This feature yield the second-largest axis length of the ROI-enclosing ellipsoid.

**DL features:** CLN_CT_DL_0, CLN_CT_DL_9, CLN_CT_DL_61, CLN_PET_DL_0, CLN_PET_DL_1, CLN_PET_DL_2, CLN_PET_DL_5, CLN_PET_DL_9, Tumor_CT_DL_3, Tumor_CT_DL_4, Tumor_CT_DL_11, Tumor_CT_DL_41, Tumor_CT_DL_57, Tumor_PET_DL_33, Tumor_PET_DL_34.
