# Supplementary material for: A 18F-FDG PET/CT-based deep learning-radiomics-clinical model for prediction of cervical lymph node metastasis in esophageal squamous cell carcinoma
Source: Cancer Imaging. 2024 Nov 12;24:153. doi: 10.1186/s40644-024-00799-0 (PMC11556142; doi:10.1186/s40644-024-00799-0)
Supplement: Supplementary file 3 — Supplementary Material 3 [file 40644_2024_799_MOESM3_ESM.docx]

**SUPPLEMENTARY FIGURE**

**TITLE: A ^18^F-FDG PET/CT-based deep learning-radiomics-clinical model for prediction of cervical lymph node metastasis in esophageal squamous cell carcinoma**

**CONTENT**

1. **Supplementary Figure 1:** The proportion of different kinds of features.
2. **Supplementary Figure 2:** Regression coefficients for LASSO are presented, with each colored line representing the curve of variation for the characteristic coefficient with respect to lambda values.
3. **Supplementary Figure 3:** The optimal λ values led to the selection of 11, 25 and 26 features for building the radiomics (A), DL (B) and DL-Radiomics (C) models, respectively; DL, deep learning.
4. **Supplementary Figure 4:** The Nomogram of DRC model.


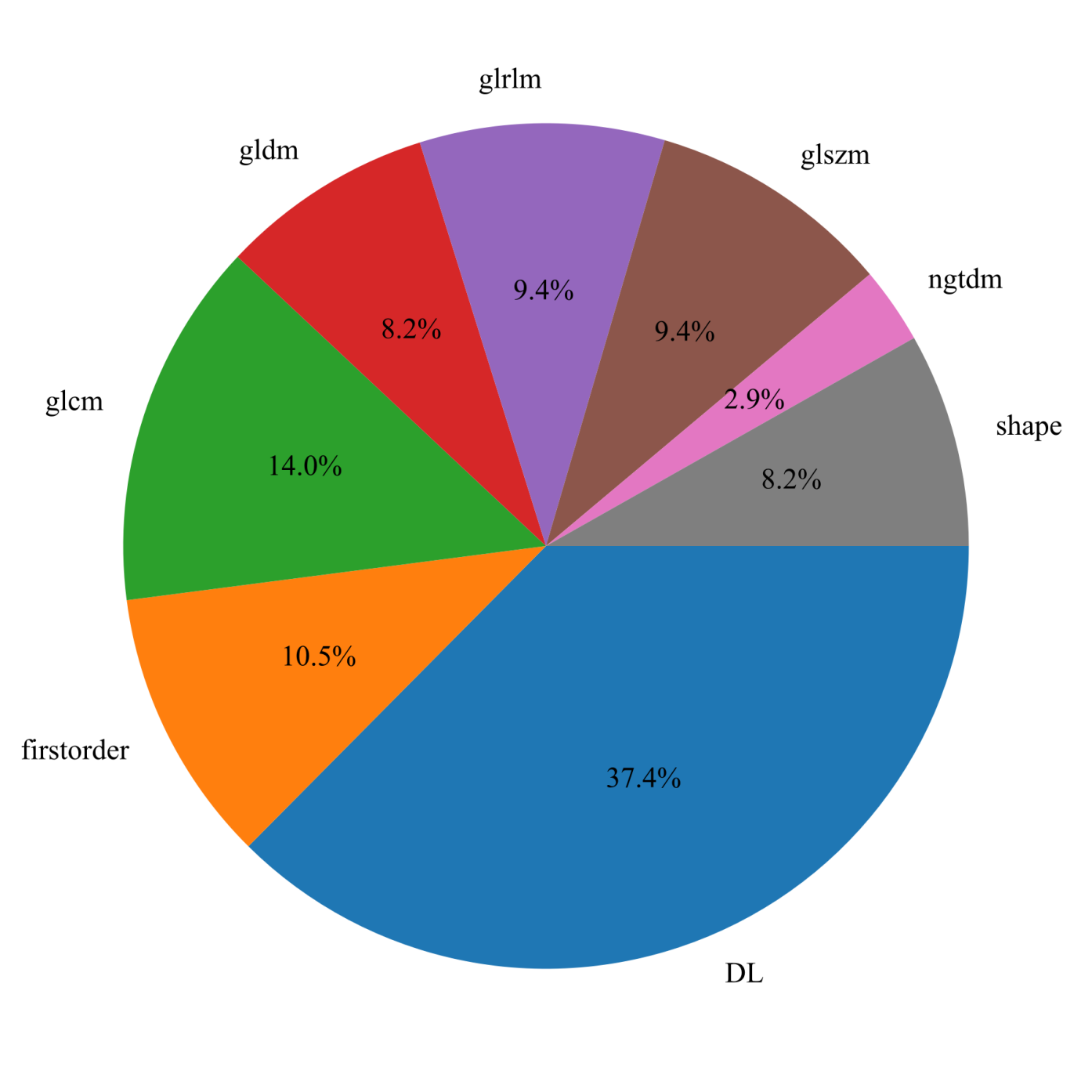


**Supplementary Figure 1:** The proportion of different kinds of features.


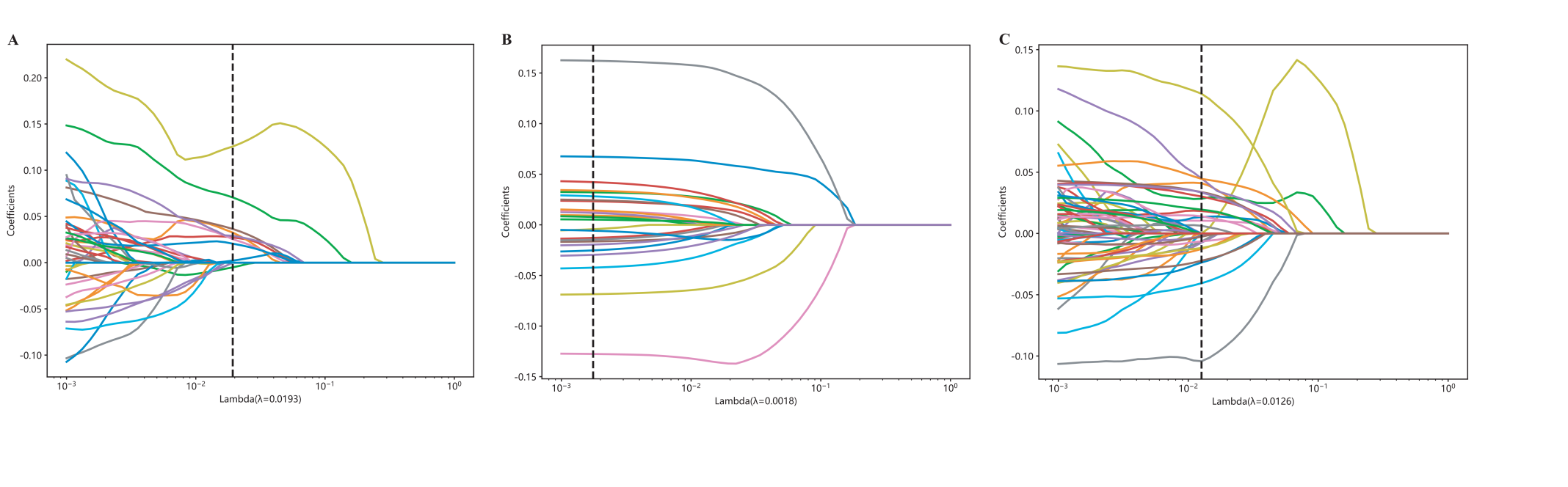


**Supplementary Figure 2:** Regression coefficients for LASSO are presented, with each colored line representing the curve of variation for the characteristic coefficient with respect to lambda values.


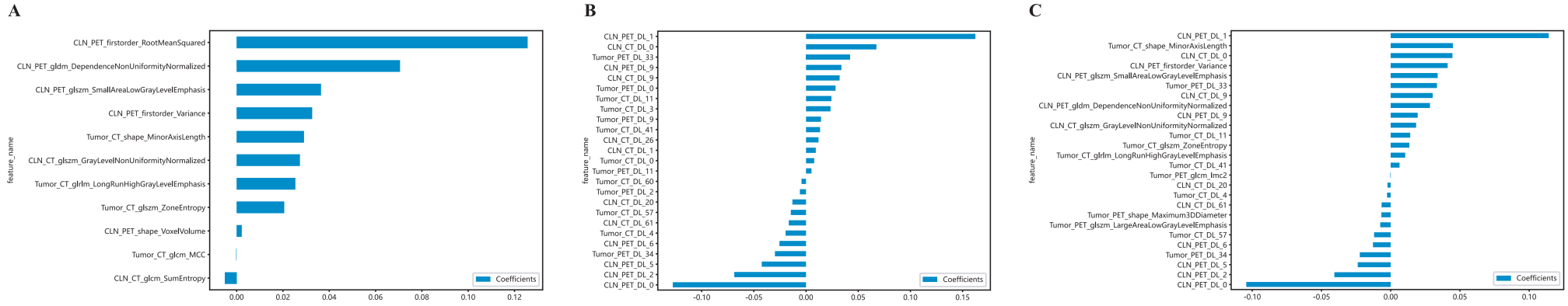


**Supplementary Figure 3:** The optimal λ values led to the selection of 11, 25 and 26 features for building the radiomics (A), DL (B) and DL-Radiomics (C) models, respectively; DL, deep learning.


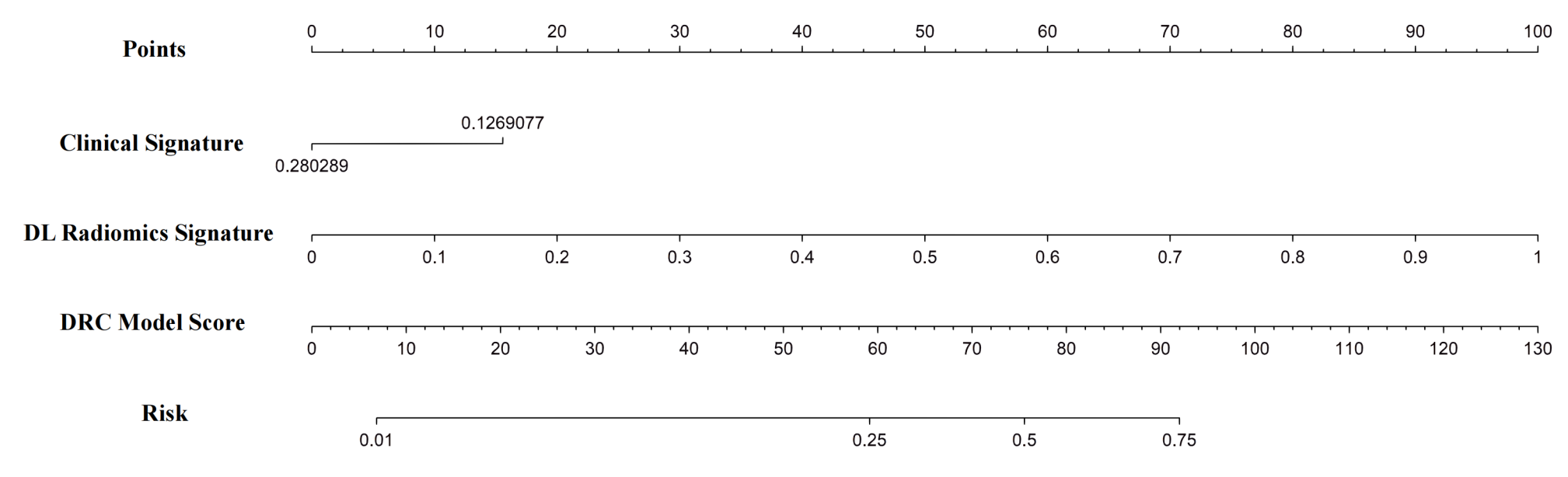


**Supplementary Figure 4:** The Nomogram of DRC model.
